# Effects of High Temperature and Nitrogen Fertilizer on the Carbon and Nitrogen Metabolism Characteristics of Rice Varieties with Differing Taste Stability

**DOI:** 10.3390/plants15071006

**Published:** 2026-03-25

**Authors:** Ke Ma, Yuanyuan Zhou, Yao Ma, Zexin Qi, Heping Xu

**Affiliations:** 1 Agronomy College, Jilin Agricultural Science and Technology College, Jilin 132101, China; 2 Agronomy College, Jilin Agricultural University, Changchun 130118, China

**Keywords:** rice, high temperatures at initial grain filling, nitrogen application rate, taste stability, grain quality

## Abstract

Temperature and nitrogen fertilizer are key environmental factors that significantly affect rice growth and grain quality. There remains a lack of systematic research on the effects of temperature and nitrogen fertilizer on carbon–nitrogen metabolism during grain-filling, and consequently on the taste quality of rice varieties with different taste characteristics. To bridge this gap, pot experiments were conducted under different temperature and nitrogen fertilizer conditions to investigate the changes in carbon and nitrogen metabolism and the quality of different high-quality and stable-taste rice varieties during the grain filling stage. Our research results indicate that high-temperature conditions inhibit both carbon and nitrogen metabolism; however, the variations differ among rice varieties with differing taste stability. Under both normal and high nitrogen levels, compared to Akita Komachi (AK), a variety with poor taste stability, Jikedao 606 (J 606), a variety with strong taste stability, maintained a certain photosynthetic capacity under high-temperature conditions, with smaller decreases in net photosynthetic rate and soil–plant analysis development values, declining by 4.30–5.59% and 4.30–5.59% respectively. The decline in the activities of nitrate reductase, glutamine synthetase, and glutamate synthase in nitrogen metabolism was relatively small; in comparison, the decrease in the activities of ADP-glucose pyrophosphorylase, granule-bound starch synthase, starch branching enzyme, and starch debranching enzyme in carbon metabolism was comparatively minor. The content of amylose and amylopectin in the grains was maintained, improving the milled rice rate and head rice rate, thereby ensuring strong stability of excellent sensory quality. Under both high-temperature and high-nitrogen conditions, the yields of the two rice varieties were maintained. In summary, variations exist in carbon and nitrogen metabolism among different rice varieties with stable excellent taste under varying temperature and nitrogen fertilizer conditions. These metabolic differences affect starch synthesis in the endosperm, ultimately influencing the stability of rice sensory quality. This study provides a theoretical basis for nitrogen fertilizer application under high-temperature conditions and the cultivation of rice varieties with excellent taste stability.

## 1. Introduction

Rice is the staple food for more than half of the world’s population [1].

High-temperature conditions severely restrict the growth and development of crops. Global temperatures are projected to rise by 1.5 °C by 2040, potentially worsening the existing challenges encountered in rice production [2]. By the end of the century, the global average surface temperature could rise by up to 4.8 °C relative to the 1986–2005 baseline [3]. Current temperatures in most rice-growing regions have already approached the optimal range for rice production. During vulnerable growth stages, increased average temperatures or the occurrence of extreme heat events exceeding threshold levels can severely hinder rice productivity [4]. The escalating impacts of climate change have increased the frequency and severity of heat stress events during rice growing seasons [5,6]. The imminent threat of high-temperature stress to rice production has attracted widespread attention from the research community [7,8].

Notably, projections indicate that by 2030, approximately 16% of global rice cultivation area may experience heat stress exceeding 5 days during the reproductive stage [9]. Research indicates that for every 1 °C increase in average temperature during the rice growing season, rice yields decrease by 6.2%, significantly impacting farmers’ income [10]. Any further increase in average temperature or transient high temperatures may be supra-optimal and could reduce grain yield. It is estimated that rice yields will decrease by 41% by the end of the 21st century [11]. Heat stress affects all aspects of plant processes, namely, germination, growth, development, reproduction, and yield [12]. The reproductive stage of rice is highly susceptible to heat stress. The results of related studies indicate that temperatures exceeding critical thresholds of 33 °C or 35 °C can severely impact rice yields, impairing spikelet fertility, shortening grain-filling duration, reducing grain weight, and compromising grain quality [13,14]. During the flowering or grain-filling stage, even short-term heat stress (>33 °C) can negatively impact rice yield [15,16]. Nevertheless, the authors of previous studies have primarily focused on heat stress during these stages, neglecting the potential impact of pre-heading high temperatures, which can also significantly affect yield formation and grain quality, with an impact level comparable to or even greater than that observed during post-heading [14,17]. For example, in one study, under similar temperature treatments, heat stress resulted in a significant reduction in grain number and filling rate during the booting stage compared to the post-heading stage [18]. Furthermore, compared to the booting and post-heading stages, heat stress during the early stage of panicle initiation has distinct effects on grain quality, particularly on head rice rate and chalkiness degree [14,18].

In another study, heat stress reduced the dry matter weight of panicles, leading to a significant decrease in assimilate allocation to grains, lower grain weight, and deterioration of rice quality, including increased chalkiness and poorer culinary and sensory qualities [13,19]. Starch and protein are the key determinants of rice sensory quality, the synthesis and accumulation of which are regulated by carbon and nitrogen metabolism during the grain-filling stage [20]. High temperatures have a significant impact on the nutritional quality of grains. For example, under high-temperature conditions, starch content decreases, whereas protein content increases [21]. Some researchers suggest that high temperatures reduce starch assimilation, leading to decreased grain length and width [22], with alterations in starch structure and arrangement contributing to increased chalkiness [23]. In addition, high temperatures during the grain-filling stage can also adversely affect the culinary and sensory quality of rice. The reduction in starch content induced by high-temperature conditions leads to higher gelatinization parameters, including changes in gelatinization temperature and gelatinization viscosity [24].

Nitrogen (N) is one of the essential nutrients required by plants and plays a crucial role in various developmental processes. Nitrogen fertilizer is one of the key factors affecting rice yield and quality. The results of other studies have demonstrated that appropriate nitrogen fertilizer management can enhance resistance to abiotic stresses such as low temperatures, high temperatures, and drought [25,26]. In one study, the increase in nitrogen fertilizer application during the flowering stage reduced the degeneration of rice spikelets and yield losses caused by high temperatures upon flowering [27]. In addition, the effect of nitrogen application on heat tolerance depends on the type and amount of nitrogen applied [27]. Optimal application of nitrogen fertilizer can further improve the physiological metabolism of rice plants under high-temperature stress and reduce heat damage [25]. The findings of other studies have demonstrated that nitrogen fertilizer application during the high-temperature heading stage significantly reduces chalkiness and increases head rice rate, which can be attributed to the delayed development of amyloplasts and the mitigation of adverse effects caused by high temperatures [28]. Exposure to high-temperature superconductivity throughout the rice growing period and after flowering reduces nitrogen allocation to grains but increases nitrogen allocation to vegetative organs [4]. Rising temperatures can increase grain nitrogen concentration, as the impact of heat stress on carbon accumulation in rice grains exceeds its effect on nitrogen [4,29]. Appropriate application of nitrogen fertilizer has been demonstrated to delay leaf senescence, enhance leaf photosynthetic efficiency, and improve plant physiological processes of carbon and nitrogen metabolism [30]. Therefore, alterations in carbon and nitrogen metabolism are crucial for the content and composition of starch and proteins in the rice endosperm, thereby impacting the overall sensory quality of rice.

Under high-temperature conditions, the transpiration rate of rice increases. High nitrogen supply enhances transpiration rate, photosynthetic rate, and root activity, mitigating the adverse effects of high temperatures on the seed setting rate and yield of rice [25]. Research findings have shown that the optimal application of nitrogen fertilizer, particularly the increased application of nitrogen fertilizer to the ear, increases the number of spikelets per ear [31]. The effect of nitrogen application on rice heat stress therefore warrants further investigation. To date, however, the authors of most studies have focused on breeding strategies aimed at mitigating the adverse effects of heat stress on grain quality and yield. Research on the relationship between different nitrogen fertilizer application rates at the initial grain filling stage and short-term heat stress on rice grain quality and yield formation remains limited. In this study, two representative rice varieties, Akita Komachi (AK) and Jikedao 606 (J 606), were subjected to heat stress conditions at the grain filling stage. The purpose of this study is to investigate the effects of nitrogen fertilizer application at the grain filling stage on rice yield, yield components, and grain quality formation under heat stress.

## 2. Results

### 2.1. Effects of Temperature and Nitrogen Fertilizer on the Photosynthetic Characteristics of Rice

**Figure 1:**
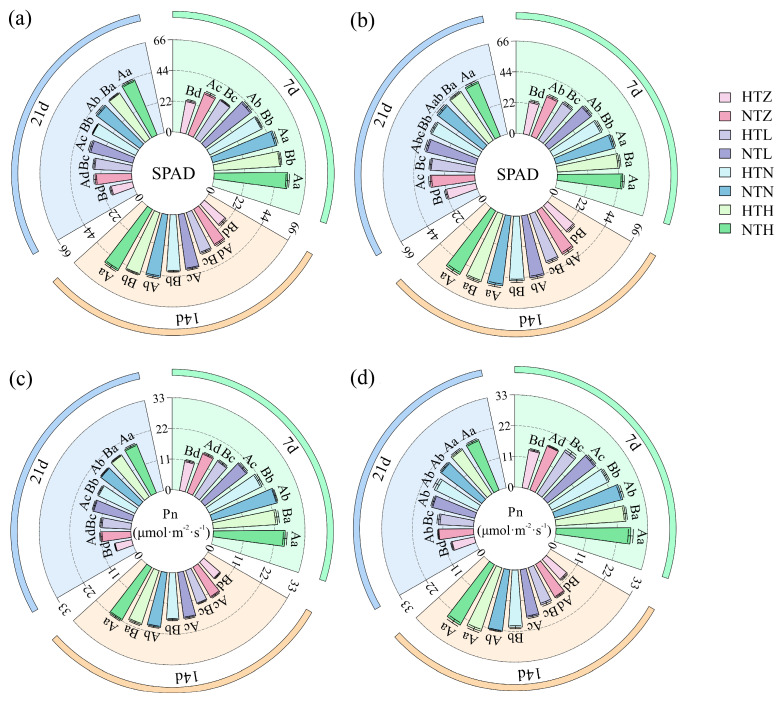
Effects of temperature and nitrogen fertilizer on photosynthetic characteristics of AK (**a**,**c**) and J 606 (**b**,**d**). Values are presented as means ± SD, n = 3. The same uppercase and lowercase letters indicate non-significance, while different uppercase/lowercase letters indicate differences at the level of *p* < 0.05.

As shown in Figure 1, different temperature and nitrogen fertilizer treatments have varying effects on rice photosynthetic characteristics. Both soil–plant analysis development (SPAD) values and net photosynthetic rate (Pn) increase with the rise in nitrogen application rate. Under normal temperature conditions, SPAD and Pn consistently remain higher than those under high-temperature treatment. During the 7–21 d treatment period, compared with NTZ, NTL, NTN, and NTH treatments, the SPAD values of AK in HTZ, HTL, HTN, and HTH treatments significantly (*p* < 0.05) decreased by an average of 33.06%, 19.45%, 11.60%, and 8.00%, respectively (Figure 1a). In the HTZ, HTL, HTN, and HTH treatments, the SPAD values of J 606 significantly (*p* < 0.05) decreased by an average of 28.42%, 16.24%, 8.65%, and 2.83%, respectively (Figure 1b). During the 7–21 d treatment period, compared with NTZ, NTL, NTN, and NTH treatments, the Pn of AK in HTZ, HTL, HTN, and HTH treatments significantly (*p* < 0.05) decreased by an average of 36.21%, 23.25%, 14.26%, and 7.21%, respectively (Figure 1c). Under HTZ, HTL, and HTN, the Pn of J 606 significantly (*p* < 0.05) decreased by an average of 26.75%, 17.90%, and 8.21%, respectively (Figure 1d).

### 2.2. Effects of Temperature and Nitrogen Fertilizer on the Carbon Metabolic Enzyme Activities in Rice

**Figure 2:**
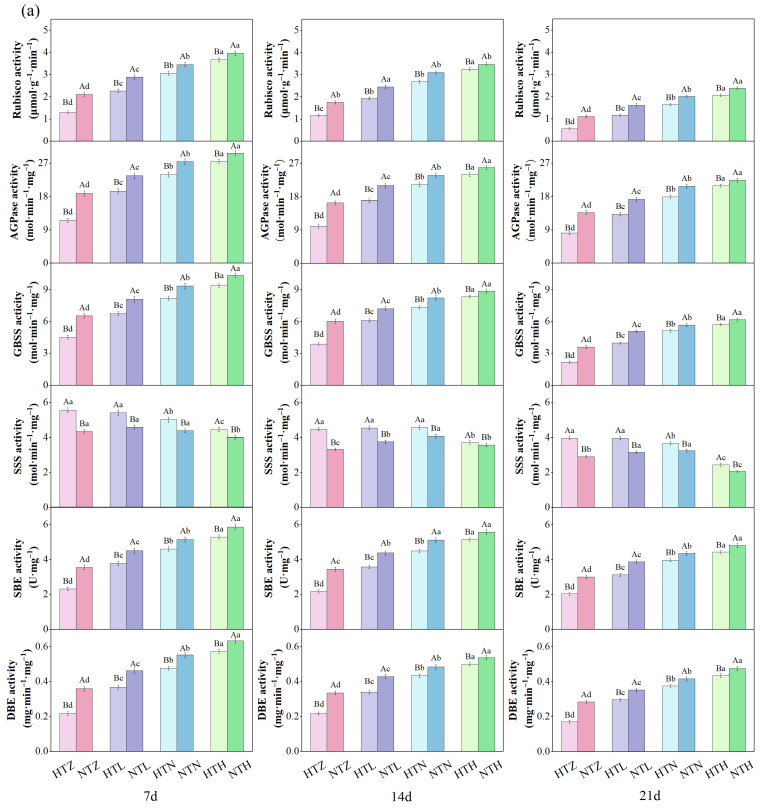

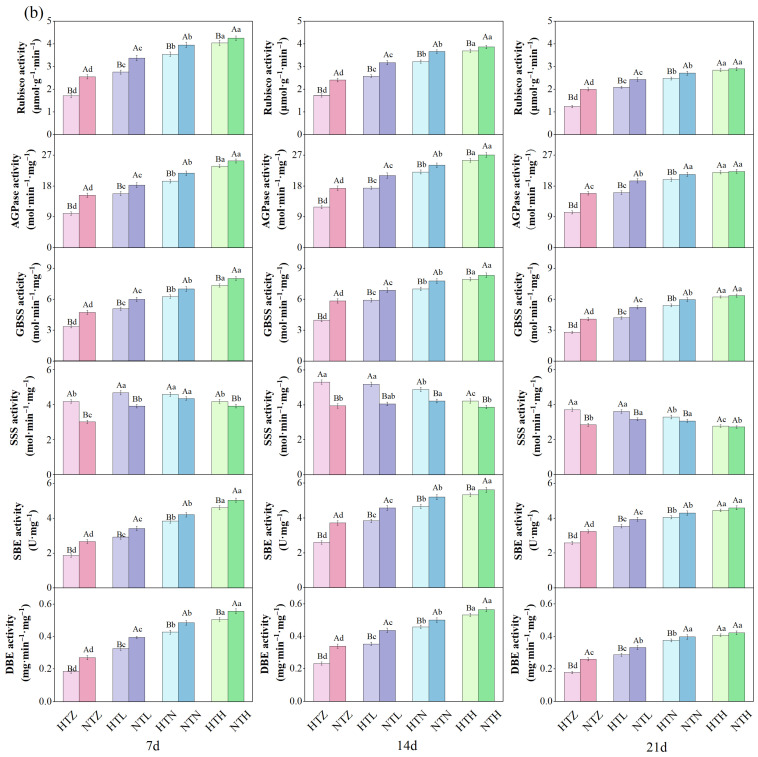
Effects of temperature and nitrogen fertilizer on carbon metabolic enzyme activities of AK (**a**) and J 606 (**b**). Values are presented as means ± SD, n = 3. The same uppercase and lowercase letters indicate non-significance, while different uppercase and lowercase letters indicate differences at the level of *p* < 0.05.

As shown in Figure 2, different temperature and nitrogen fertilizer treatments affected the activities of carbon metabolic enzymes in rice grains. The activities of ribulose-1,5-bisphosphate carboxylase/oxygenase (Rubisco), ADP-glucose pyrophosphorylase (AGPase), granule-bound starch synthase (GBSS), starch branching enzyme (SBE), and starch debranching enzyme (DBE) in both rice grains increased with the increase of nitrogen application rate. In contrast, the activity of soluble starch synthase (SSS) showed an opposite trend. During the 7–21 d treatment period, compared with the NTZ, NTL, NTN, and NTH treatments, the Rubisco activity of AK in the HTZ, HTL, HTN, and HTH treatments significantly (*p* < 0.05) decreased by an average of 40.39%, 23.43%, 14.41%, and 9.07%, respectively; AGPase activity significantly (*p* < 0.05) decreased by an average of 39.18%, 20.09%, 12.27%, and 7.12%; GBSS activity significantly (*p* < 0.05) decreased by an average of 35.54%, 17.95%, 11.00%, and 7.53%; SBE activity significantly (*p* < 0.05) decreased by an average of 34.66%, 17.86%, 10.51%, and 8.36%; and DBE activity significantly (*p* < 0.05) decreased by an average of 38.16%, 19.19%, 11.29%, and 8.46%; while SSS activity significantly (*p* < 0.05) increased by an average of 24.69%, 17.57%, 11.70%, and 9.67%, respectively. Meanwhile, in J 606, the activities of Rubisco in the HTZ, HTL, and HTN treatments significantly (*p* < 0.05) decreased by an average of 33.14%, 17.09%, 10.41%, and 3.64%, respectively; AGPase activities significantly (*p* < 0.05) decreased by an average of 33.69%, 16.09%, 8.65%, and 4.32%; GBSS activities significantly (*p* < 0.05) decreased by an average of 30.70%, 16.22%, 9.86%, and 4.95%; SBE activities significantly (*p* < 0.05) decreased by an average of 26.74%, 13.61%, 8.26%, and 5.57%; DBE activities significantly (*p* < 0.05) decreased by an average of 31.45%, 16.63%, 8.63%, and 6.15%; and SSS activities significantly (*p* < 0.05) increased by an average of 25.69%, 16.91%, 8.51%, and 5.46%.

### 2.3. Effects of Temperature and Nitrogen Fertilizer on the Nitrogen Metabolism Enzyme Activities of Rice

**Figure 3:**
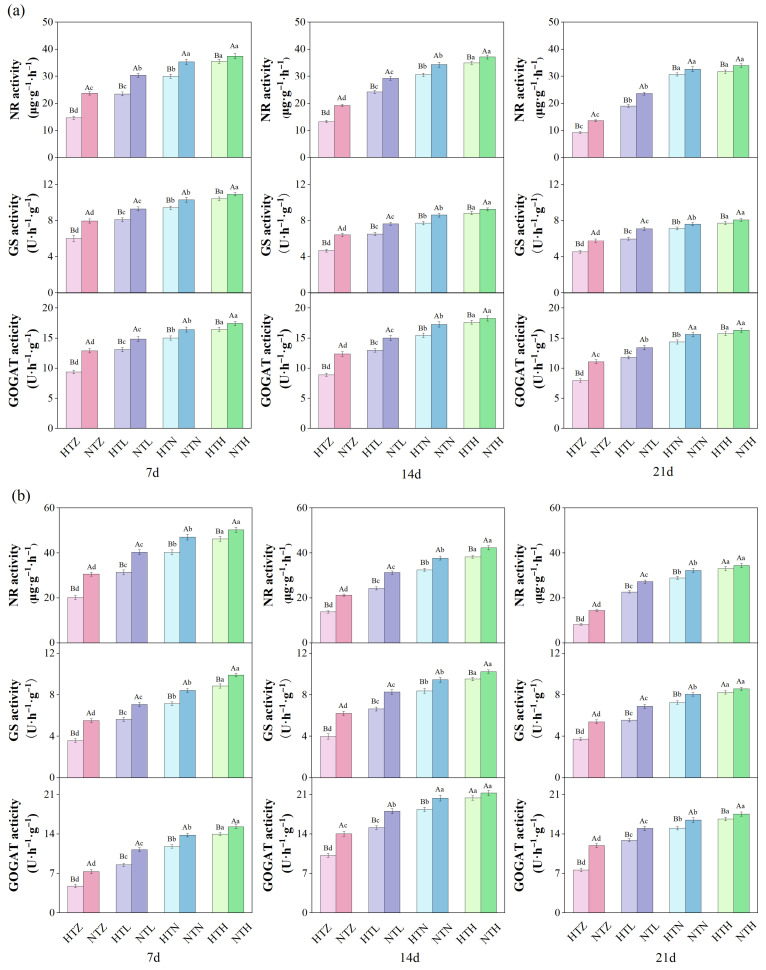
Effects of temperature and nitrogen fertilizer on nitrogen metabolism enzyme activities of AK (**a**) and J 606 (**b**). Values are presented as means ± SD, n = 3. The same uppercase and lowercase letters indicate non-significance, while different uppercase and lowercase letters indicate differences at the level of *p* < 0.05.

Figure 3 results indicate that different temperature and nitrogen fertilizer treatments can significantly alter the activities of nitrogen metabolism enzymes nitrate reductase (NR), glutamine synthetase (GS), and glutamate synthase (GOGAT) in rice. Under normal temperature conditions, within the same time period, increasing nitrogen application significantly enhanced NR activity, while the activities of GS and GOGAT showed relatively smaller increases with higher nitrogen levels. During the 7–21 d treatment period, compared to the NTZ, NTL, NTN, and NTH treatments, the average NR of AK under the HTZ, HTL, HTN, and HTH treatments significantly (*p* < 0.05) decreased by 37.26%, 20.47%, 10.66%, and 7.26%, respectively; GS significantly (*p* < 0.05) decreased by 34.00%, 19.83%, 12.10%, and 7.20%, respectively; and GOGAT significantly (*p* < 0.05) decreased by 32.76%, 18.11%, 11.06%, and 5.92%, respectively (Figure 3a). Compared with the NTZ, NTL, NTN, and NTH treatments, the average NR of J 606 under the HTZ, HTL, and HTN treatments significantly (*p* < 0.05) decreased by 33.75%, 19.74%, and 12.84%, respectively; GS significantly (*p* < 0.05) decreased by 24.19%, 14.61%, and 8.49%, respectively; and GOGAT significantly (*p* < 0.05) decreased by 27.89%, 12.55%, and 8.96%, respectively (Figure 3b).

### 2.4. Effects of Temperature and Nitrogen Fertilizer on the Starch Content in Rice Grains

**Figure 4:**
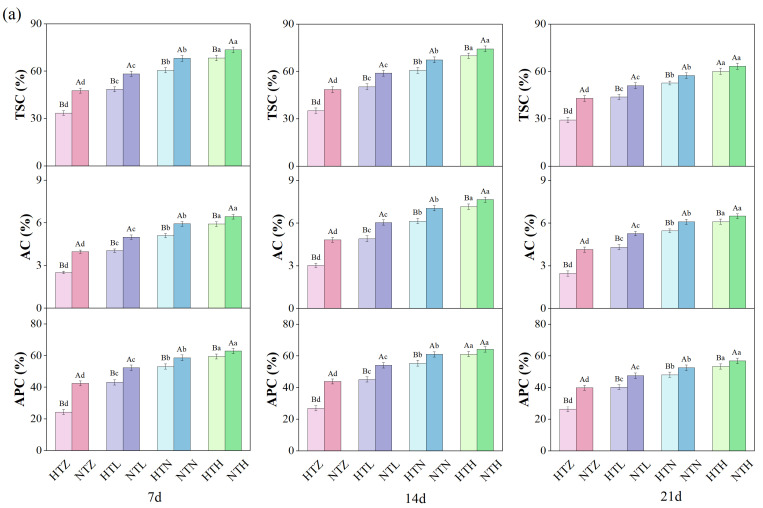

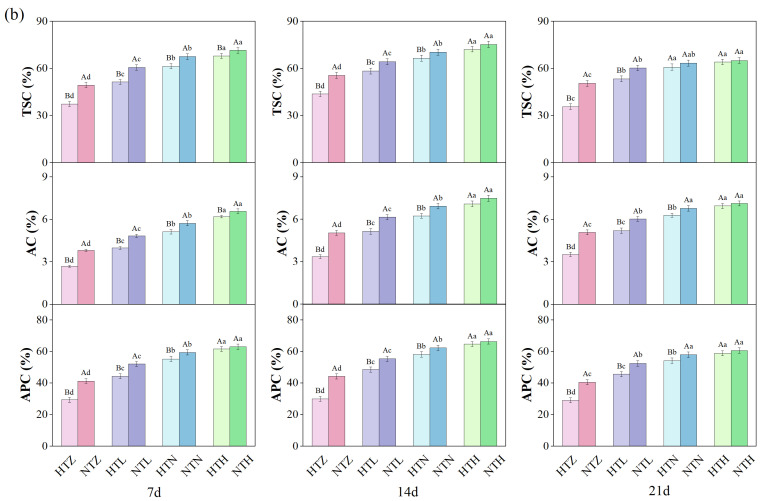
Effects of temperature and nitrogen fertilizer on starch content of AK (**a**) and J 606 (**b**). Values are presented as means ± SD, n = 3. The same uppercase and lowercase letters indicate non-significance, while different uppercase and lowercase letters indicate differences at the level of *p* < 0.05.

As shown in Figure 4, different nitrogen fertilizer treatments had significant affects on rice starch content under high-temperature conditions. Under the same duration of high-temperature treatment, total starch content (TSC), amylose content (AC), and amylopectin content (APC) increased with the increase of nitrogen application rate. After 7 days of treatment, compared with the NTZ, NTL, NTN, and NTH treatments, the TSC activity of AK under the HTZ, HTL, HTN, and HTH treatments significantly (*p* < 0.05) decreased by 29.73%, 16.64%, 11.15%, and 7.17%, respectively; the AC activity significantly (*p* < 0.05) decreased by 36.74%, 19.02%, 13.97%, and 8.09%, respectively; and the APC activity significantly (*p* < 0.05) decreased by 42.69%, 17.76%, 9.48%, and 5.49%, respectively (Figure 4a). The J 606 cultivar showed similar trends in enzyme activity changes. Compared with the NTZ, NTL, NTN, and NTH treatments, the TSC activity under the HTZ, HTL, HTN, and HTH treatments significantly (*p* < 0.05) decreased by 24.29%, 15.25%, 9.11%, and 4.99%, respectively; the AC activity significantly (*p* < 0.05) decreased by 29.49%, 17.65%, 10.59%, and 5.78% (Figure 4b).

After 21 days of treatment, compared with the NTZ, NTL, and NTN treatments, the TSC activity of AK under the HTZ, HTL, and HTN treatments significantly (*p* < 0.05) decreased by 31.90%, 14.16%, and 8.23%, respectively; the AC activity significantly (*p* < 0.05) decreased by 40.52%, 18.24%, and 10.41%, respectively; and the APC activity significantly (*p* < 0.05) decreased by 33.82%, 15.39%, and 8.51%, respectively (Figure 4a). Compared with the NTZ, NTL, and NTN treatments, the TSC activity of J 606 under the HTZ, HTL, and HTN treatments significantly (*p* < 0.05) decreased by 29.32%, 11.37%, and 4.01%, respectively; the AC activity significantly (*p* < 0.05) decreased by 30.67%, 13.72%, and 7.27%, respectively; and the APC activity significantly (*p* < 0.05) decreased by 28.28%, 13.13%, and 6.55%, respectively (Figure 4b).

### 2.5. Effects of Temperature and Nitrogen Fertilizer on the Nitrogen Content in Rice Grains

**Figure 5:**
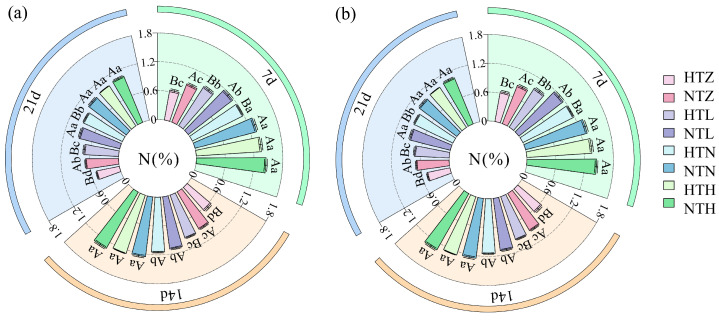
Effects of temperature and nitrogen fertilizer on nitrogen content of AK (**a**) and J 606 (**b**). Values are presented as means ± SD, n = 3. The same uppercase and lowercase letters indicate non-significance, while different uppercase and lowercase letters indicate differences at the level of *p* < 0.05.

As shown in Figure 5, both temperature and nitrogen fertilizer effected changes in the N content of rice grains, with different varieties exhibiting varying trends. During the 7–21 d treatment period, compared with the NTZ, NTL, and NTN treatments, AK showed significant (*p* < 0.05) average reductions of 33.25%, 20.06%, and 13.48% in N content under the HTZ, HTL, HTN, and HTH treatments, respectively (Figure 5a). Compared with the NTZ, NTL, and NTN treatments, J 606 showed, on average, significant (*p* < 0.05) decreases of 27.59%, 15.89%, and 9.59% in N content under the HTZ, HTL, HTN, and HTH treatments, respectively (Figure 5b).

### 2.6. Effects of Temperature and Nitrogen Fertilizer on the Rice Yield and Components

As shown in Table 1, nitrogen fertilizer primarily affected the number of grains per panicle, 1000-grain weight, and yield of the two rice varieties. Under the same nitrogen application level, the high-temperature treatment mainly influenced the 1000-grain weight of AK. Compared with the HTZ, HTL, HTN, and HTH treatments, AK increased the 1000-grain weight by 12.60%, 16.32%, 9.91%, and 14.08% respectively in the NTZ, NTL, NTN, and NTH treatments, while the yield increased by 6.23%, 4.79%, 5.69%, and 4.40% respectively. Meanwhile, the yields of J 606 in the NTZ, NTL, NTN, and NTH treatments increased by 5.10%, 3.93%, 4.12%, and 3.72% respectively. Under ambient temperature conditions, compared with the NTZ treatment, AK showed an increase in grains per panicle of 11.99–33.12%, a decrease in 1000-grain weight of −14.88–6.98%, and an increase in yield of 23.53–51.87%. For J 606, the grains per panicle increased by 3.19–18.61%, 1000-grain weight decreased by −9.24–−0.84%, and yield increased by 29.33–51.84%.

### 2.7. Effects of Temperature and Nitrogen Fertilizer on the Rice Taste Value

**Figure 6:**
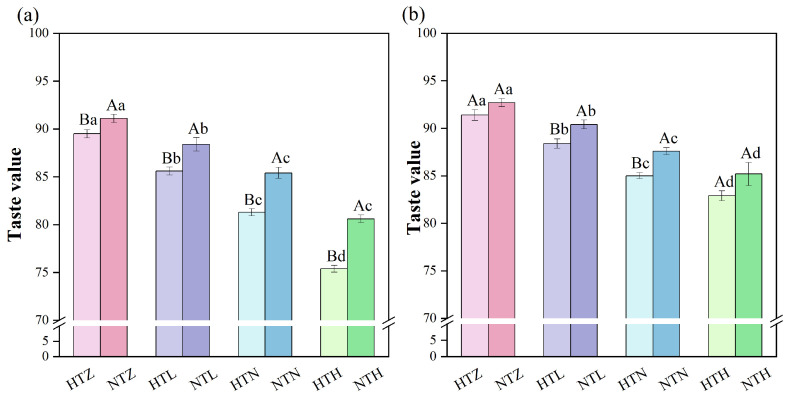
Effects of temperature and nitrogen fertilizer on the taste value of AK (**a**) and J 606 (**b**). Values are presented as means ± SD, n = 3. The same uppercase and lowercase letters indicate non-significance, while different uppercase and lowercase letters indicate differences at the level of *p* < 0.05.

As shown in Figure 6, the taste value was negatively correlated with nitrogen application rate. Under the same nitrogen application condition, the taste value at normal temperatures was significantly higher than that at low temperatures. Under room temperature conditions, compared with zero N, the taste value of AK significantly (*p* < 0.05) decreased by 3.28–9.30%, while that of J 606 significantly (*p* < 0.05) decreased by 4.36–15.75%. Compared with the NTZ, NTL, NTN, and NTH treatments, the taste values of AK under the HTZ, HTL, HTN, and HTH treatments significantly (*p* < 0.05) decreased by 1.75%, 3.17%, 4.80%, and 6.45%, respectively. The taste values of J 606 decreased by 1.40%, 2.21%, 2.97%, and 2.70%, respectively.

### 2.8. Effects of Temperature and Nitrogen Fertilizer on the Appearance Quality and Processing Quality of Rice

As shown in Table 2, nitrogen fertilizer had effects on the head rice rate, brown rice rate, milled rice rate, chalkiness degree, and chalky grain percentage of the two rice varieties. Among these, the head rice rate, brown rice rate, and milled rice rate increased with the rise in the nitrogen application rate, while the chalkiness degree and chalky grain percentage exhibited opposite trends. Under the same nitrogen fertilization level, the high-temperature treatment had varying degrees of impact on quality indicators. Compared with the HTZ, HTL, HTN, and HTH treatments, AK in the NTZ, NTL, NTN, and NTH treatments showed the following improvements: the brown rice rate increased by 1.15%, 1.89%, 1.60%, and 2.15% respectively; the milled rice rate increased by 0.75%, 1.19%, 1.73%, and 1.40% respectively; the head rice rate increased by 2.01%, 3.20%, 3.95%, and 3.77% respectively; the chalkiness degree decreased by 12.50%, 11.11%, 9.10%, and 5.13% respectively; and the chalky grain rate decreased by 3.69%, 1.84%, 2.87%, and 3.30% respectively. Meanwhile, for J 606 under the NTZ, NTL, NTN, and NTH treatments, the brown rice rate increased by 2.00%, 2.94%, 2.62%, and 2.53% respectively; the milled rice rate increased by 0.44%, 1.75%, 1.53%, and 1.64% respectively; the head rice rate increased by 2.53%, 4.01%, 4.80%, and 4.13% respectively; the chalkiness degree decreased by 9.62%, 7.95%, 4.29%, and 2.70% respectively; and the chalky grain rate decreased by 5.10%, 2.92%, 2.08%, and 1.27% respectively.

### 2.9. Correlation Analysis

**Figure 7:**
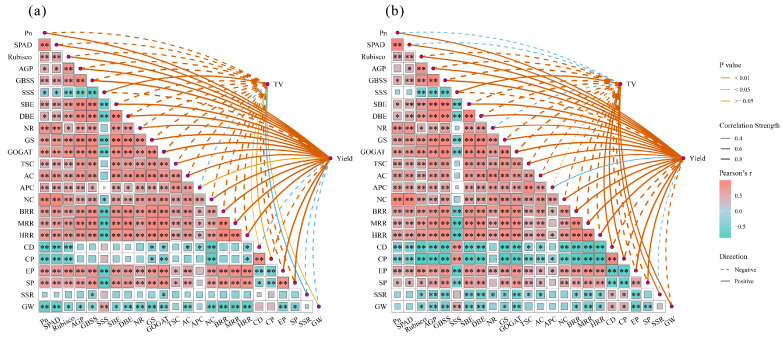
Correlation analysis of various indicators between AK (**a**) and J 606 (**b**). * indicates *p* < 0.05, ** indicates *p* < 0.01. Note: TV: taste value; BRR: HRR: head rice rate; BRR: brown rice rate; MRR: milled rice rate; CD: chalkiness degree; CP: chalky grain percentage; EP: effective panicle number; SP: spikelets per panicle; SSR: seed setting rate; GW: 1000-grain weight.

In AK, TV showed a highly significant negative correlation with Pn, SPAD, and NR, and a significant positive correlation with SSR (Figure 7a). Yield was significantly negatively correlated with CD, SSR, and GW. In J 606, TV exhibited a significant negative correlation with Pn, SPAD, and NR, and a highly significant positive correlation with SSS, CD, CP, SSR, and GW (Figure 7b). Yield was significantly positively correlated with APC, while showing highly significant negative correlations with CD, CP, and GW, and a significant negative correlation with SSR.

### 2.10. Principal Component Analysis

**Figure 8:**
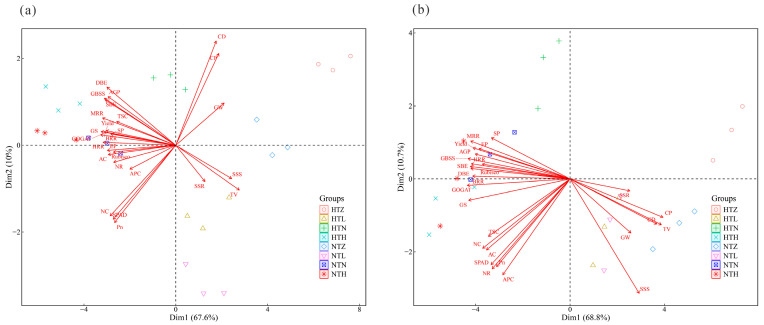
Principal component analysis of various indicators for AK (**a**) and J 606 (**b**).

In AK, the first two axes of the principal component analysis (PCA) explained 77.6% of the total observed variation. Principal component 1 (67.6%) was associated with TV, SSS, and GW, while principal component 2 (10.0%) was associated with CD, CP, and DBE (Figure 8a). In J 606, the first two axes of the principal component analysis (PCA) explained 79.5% of the total observed variation (Figure 8b). Principal component 1 (68.8%) was associated with TV, CP, and CD, while principal component 2 (10.0%) was associated with SSS, APC, and NR.

## 3. Discussion

Nitrogen is one of the most essential nutrients for rice growth. Nitrogen application is a crucial cultivation measure for regulating rice growth and development. Optimal nitrogen fertilization can enhance the physiological metabolism level of rice plants under high-temperature stress, thereby mitigating heat damage [32]. Nitrogen contributes to the composition of photosynthetic pigments, photosynthetic enzymes, and chloroplasts. The nitrogen content in leaves has a positive effect on photosynthesis [33,34]. In one study, nitrogen application enhanced the physiological metabolic level of rice plants under high temperatures. In some treatments, stomatal conductance increased, leaf stomatal resistance decreased, and transpiration rate rose, facilitating CO_2_ uptake and water vapor release through stomata. The water vapor absorbed heat during evaporation, leading to a decrease in the temperature of the panicles and flag leaves. Consequently, the inhibition of photosynthesis under high temperatures was partially alleviated, thereby improving the net photosynthetic rate of the flag leaves [25]. In this study, with the increase in nitrogen fertilizer application rate, the SPAD and Pn of rice leaves exhibited an increasing trend. After high-temperature treatment, the SPAD and Pn of rice leaves under zero-nitrogen and low-nitrogen conditions decreased significantly, with the decrease being less significant under normal-nitrogen and high-nitrogen conditions. Under high-temperature conditions, nitrogen application, particularly normal nitrogen and high nitrogen levels, alleviates damage to the photosynthetic system and mitigates the negative effects of high temperatures on rice leaf photosynthesis, thereby ensuring the supply of assimilates required for grain filling. Under both normal- and high-nitrogen conditions, compared with AK, cultivar J 606 exhibited relatively smaller decreases in SPAD and Pn after high-temperature treatment. The changes in the correlation between taste value and SPAD or Pn also indicated that varieties with stable taste value exhibited weakened negative correlation with photosynthesis.

The results of previous studies have demonstrated that, in addition to rice genotype, environmental factors such as nitrogen fertilizer, temperature, and drought can also significantly affect carbon and nitrogen metabolism in rice [35,36,37]. In this study, we observed that with an increase in the nitrogen application rate, the activities of Rubisco, AGPase, GBSS, SBE, and DBE in both rice varieties exhibited an upward trend. Compared with high-temperature treatment, normal nitrogen and high nitrogen levels alleviated the activities of Rubisco, AGPase, GBSS, SBE, and DBE in rice, and the difference between the two decreased with treatment time. High-temperature treatment increased SSS activity, with the addition of nitrogen fertilizer resulting in a decrease in SSS activity. The activity of Rubisco in leaves exhibited a consistent trend with the changes in Pn, and the decline in Pn led to a decrease in the capacity to assimilate CO_2_. The results of related studies have demonstrated that under unfavorable conditions, the activities of starch synthase enzymes in rice grains decrease, including AGPase, GBSS, SBE, and DBE activities [38,39]. Under normal- and high-nitrogen conditions, compared with AK, J 606 exhibited relatively less pronounced decreases in Rubisco, AGPase, GBSS, SBE, and DBE after high-temperature treatment.

In plants, some photosynthetic products are primarily retained in the form of sucrose and starch, which are used for leaf respiration and the maintenance of sucrose export overnight [40]. Different species exhibit variations in carbohydrate accumulation in their leaves; some varieties primarily accumulate sucrose, whereas others mainly accumulate starch [41]. Starch composition is a crucial determinant of rice quality, as starch constitutes the largest proportion of its components [42]. In this study, the contents of amylose, amylopectin, and total starch in the two rice varieties increased with the increase in nitrogen application rate, with high-temperature treatment further reducing the contents of amylose, amylopectin, and total starch. These results are consistent with previous research findings [43]. The starch content increased with the rise in the nitrogen application rate, primarily due to the enhanced activity of starch synthase in rice grains. The activities of Rubisco, AGPase, GBSS, SBE, and DBE further promoted the synthesis of amylose, amylopectin, and total starch. Under both normal and high-nitrogen conditions, compared with AK, J 606 exhibited relatively smaller decreases in amylose, amylopectin, and total starch content after high-temperature treatment. In summary, varieties with stable taste values exhibit relatively smaller declines in carbon metabolism enzyme activity and starch content under high-temperature conditions.

Nitrogen fertilization is a key factor influencing nitrogen metabolism. The results of related studies indicate that increasing nitrogen application can slow down chlorophyll degradation, thereby delaying leaf senescence [44]. Nitrogen metabolism plays a crucial role in plant growth and development. NR is the rate-limiting enzyme for NO_3_^−^ assimilation. As NO_3_^−^ assimilation is an energy-intensive process [45], NR activity serves as the primary controlling factor for nitrate uptake rates [46,47,48]. GS, as a key enzyme involved in plant NH_4_^+^ assimilation, is the critical enzyme that incorporates NH_4_^+^ into amides and amino acids. Inorganic nitrogen must be assimilated into organic nitrogen before it can be absorbed and utilized, with GOGAT being the most important assimilatory metabolite for ammonia synthesis [49,50,51,52,53]. In this study, the activities of NR, GS, and GOGAT in the two rice cultivars increased with increasing nitrogen application rates, with high-temperature treatment further reducing the activities of NR, GS, and GOGAT. Under normal-nitrogen conditions, after high-temperature treatment, the average decrease rates of NR, GS, and GOGAT activities in AK were 11.50%, 12.10%, and 11.06%, respectively; in comparison, those in J 606 were 12.36%, 8.49%, and 8.96%, respectively. Under high-nitrogen conditions, the average decrease rates of AK were 7.26%, 7.20%, and 5.92%, respectively; in comparison, those of 654 were 5.84%, 4.41%, and 4.24%, respectively. In summary, under both the normal- and high-nitrogen conditions, compared with AK, J 606 exhibited a relatively smaller decline in nitrogen metabolism enzyme activity after high-temperature treatment. The changes in nitrogen content in the grains further validated nitrogen assimilation in nitrogen metabolism.

Rice quality is also affected by nitrogen fertilizer and environmental conditions. Carbon and nitrogen metabolism are closely related to rice sensory quality. Through the regulation of carbon and nitrogen substrates and enzymes, they collectively influence starch synthesis in the endosperm, thereby affecting the sensory quality of rice [43]. In this study, our results showed that increasing nitrogen fertilizer application can improve rice processing quality, such as the brown rice rate, milled rice rate, and head rice rate, which is consistent with the results of numerous studies [54]. The improvement may be attributed to enhanced nitrogen metabolism, which increases nutrient content in grains and elevates grain hardness, thereby improving rice’s resistance to milling. This factor significantly increases the brown rice rate, milled rice rate, and head rice rate [55]. However, differences in cultivar type and environmental conditions may lead to differing research outcomes. In this study, under the same nitrogen fertilizer level, high-temperature treatment further reduced the brown rice rate, milled rice rate, and head rice rate, with consistent trends observed in both AK and J 606. Under normal and high-nitrogen conditions, compared with AK, J 606 exhibited a relatively smaller decrease in the head rice rate after high-temperature treatment.

Conclusions vary regarding the effect of nitrogen on the appearance quality of rice. The results of some studies indicate that the chalky grain rate and chalkiness degree of rice increase with higher nitrogen application rates [56,57]. However, research findings also indicate that with the increase in nitrogen fertilizer application, the chalky grain rate and chalkiness degree of rice decrease [58]. In a study by Wang et al., the authors showed that with an increase in nitrogen levels, the chalky grain rate and chalkiness degree first decreased and then increased [59]. In a study by Guo et al., the authors demonstrated that appropriate application of nitrogen fertilizer can improve the appearance quality of rice [60]. These findings can be explained by the fact that appropriate application of nitrogen fertilizer can prolong the grain-filling period, reduce the grain-filling rate, and provide a material guarantee for grain filling, thereby reducing the formation of chalkiness in rice. In this study, the chalkiness degree and chalky grain rate of rice first decreased and then increased with an increase in nitrogen fertilizer. Under the same nitrogen application level, high-temperature treatment further increased the chalkiness degree and chalky grain rate. Under normal and high-nitrogen conditions, compared with AK, J 606 exhibited relatively smaller increases in chalkiness degree and chalky grain rate after high-temperature treatment. Therefore, rice varieties with stable taste values exhibit relatively consistent processing quality and appearance quality under both normal-nitrogen and high-nitrogen treatments.

Environmental conditions and management practices play a crucial role in yield and yield components. Nitrogen fertilizer application is an important management measure in rice cultivation. Optimal nitrogen fertilizer management, including appropriate application rates, can enhance crop yields. The results of related studies [61] indicate that yield and quality losses in rice are closely associated with the accelerated senescence of rice vegetative organs under high temperatures. It can therefore be concluded that supplementing nitrogen fertilizer during the late growth stage of rice can mitigate the damage caused by high temperatures. In this study, high-temperature treatment reduced rice yield by decreasing the number of panicles per pot and grains per panicle, although the effects were not statistically significant. High temperature and nitrogen application measures had no significant effect on the number of spikes per pot, as this spike count had already been established before heading. In this study, both temperature and nitrogen treatments were applied from the grain filling stage, omitting the spikelet formation period from jointing to heading. Therefore, under the same nitrogen level, high temperatures did not significantly affect the number of grains per panicle. High-temperature treatment further reduced the seed-setting rate and 1000-grain weight. This result is consistent with findings from other studies indicating that high temperatures decrease the seed-setting rate [62]. However, under high-temperature conditions, rice yield did not significantly decrease under the high-nitrogen treatment. Therefore, the addition of nitrogen fertilizer further alleviated the damage caused by high temperature on rice yield formation and helped maintain rice yield.

## 4. Materials and Methods

### 4.1. Test Materials

In the preliminary stage of this study, high-quality-tasting rice germplasms were screened through different nitrogen fertilizer gradients. Among them, two varieties were selected as experimental materials: Akita Komachi (AK), which exhibits excellent taste value but relatively poor stability, and Jikedao 606 (J 606), which demonstrates both exceptional taste value and strong stability.

### 4.2. Experimental Design

The pot experiment was conducted from April to October 2025 during the rice growing season in the greenhouse of Jilin Agricultural Science and Technology College, China. Pre-germination treatment was conducted on 8 April. Rice seeds were sterilized with 30% (volume fraction) sodium hypochlorite solution for 30 min, then rinsed with distilled water. They were exposed to 35 °C in darkness for 2 days to break seed dormancy. After germination, the seeds were sown in seedling trays. On 25 May, the rice seedlings were transplanted with three seedlings per plastic pot. The soil used in the experiment was taken from the upper 20 cm layer of a rice field at the Experimental Farm of Jilin Agricultural Science and Technology College. The soil was classified as sandy loam according to USDA Soil Taxonomy. The soil properties are as follows: pH 6.7, organic matter 24.21 g·kg^−1^, total nitrogen 1.93 g·kg^−1^, available nitrogen 135.12 mg·kg^−1^, available phosphorus 26.02 mg·kg^−1^, and available potassium 164.24 mg·kg^−1^.

The soil was air-dried, sieved, mixed, and then used to fill plastic pots (30 cm in height, 25 cm in diameter) with a weight of 13 kg pot^−1^. One week before transplanting rice seedlings, soak the potted soil with water until it is completely saturated. A water depth of 3 cm above the soil surface was maintained during the experimental period. Using urea (46%) as the nitrogen source, apply in three batches. Among them, 0.64 g nitrogen fertilizer per pot was applied as basal fertilizer, and 0.48 g nitrogen fertilizer was used as topdressing at 12 days after transplanting. The application of panicle fertilizer was set at four levels: no nitrogen application during the entire growth period (0 g), low panicle fertilizer application (0.34 g), conventional panicle fertilizer application (0.48 g), and high panicle fertilizer application (0.62 g). Among them, 0.37 g of phosphate fertilizer (P_2_O_5_ 12%) and 0.49 g of potassium fertilizer (K_2_O 50%) were applied as basal fertilizer in a single application.

The development of young panicles was assessed using the leaf age index or residual method [32]. Rice plants grow under natural conditions until the initial heading stage begins, which is marked by the visibility of the first row of floral primordia at the shoot tip. Subsequently, during the late booting stage of rice, the potted plants were placed under high-temperature stress conditions in the artificial climate chamber. After 21 days of treatment, the pots were returned to the natural environment until harvest. The heat stress treatment was conducted in two independent temperature-controlled chambers (Zhizhong Company, Beijing, China). The rice plants were grown under controlled conditions, including 60% relative humidity, 12 h of light (10,000 lx) from 6:00 AM to 6:00 PM daily, and 12 h of darkness from 6:00 PM to 6:00 AM. This experiment employed two treatments: a control treatment exposed to normal temperatures (NT, day/night: 28 °C/18 °C) and a high-temperature treatment (HT, day/night: 34 °C/24 °C). This experiment consisted of eight treatments, with the meaning of each treatment shown in Table 3. Each treatment had three replicates, with 20 pots per replicate. During the rice growing season, the occurrence of pests, diseases, and weeds was strictly controlled.

### 4.3. Measurement Indicators and Methods

#### 4.3.1. Measurement of Photosynthetic Characteristics and Soil–Plant Analysis Development (SPAD) Values (SPAD)

At 7, 14, and 21 days of treatment, rice plants with basically identical growth conditions were selected, and each treatment was measured in triplicate. The net photosynthetic rate (Pn) was measured from 8:00 to 11:00 AM using a portable photosynthesis system (Li-6800, Li-COR, Lincoln, NE, USA). We set the light intensity in the leaf chamber to 1200 µmol·m^−2^ s^−1^. The SPAD value of the uppermost fully expanded leaf of rice was measured using a chlorophyll meter (SPAD-502, Minolta Camera Co., Osaka, Japan).

#### 4.3.2. Determination of Carbon and Nitrogen Metabolic Enzyme Activities

At 7, 14, and 21 days of treatment, rice plants with essentially identical growth conditions were selected, with three replicate measurements conducted for each treatment. We froze the leaf and grain samples immediately and stored them at −70 °C for subsequent enzyme analysis. After grain dehulling, the activities of ADP-glucose pyrophosphorylase (AGPase), granule-bound starch synthase (GBSS), soluble starch synthase (SSS), starch branching enzyme (SBE), starch debranching enzyme (DBE), glutamine synthetase (GS), and glutamate synthase (GOGAT) were evaluated separately. After removing the midrib from the sword leaves, the activities of ribulose-1,5-bisphosphate carboxylase/oxygenase (Rubisco) and nitrate reductase (NR) were measured. All enzyme activities were measured according to the protocols outlined in the corresponding kits provided by Shanghai Enzyme-linked Biotechnology Co., Ltd., Shanghai, China.

#### 4.3.3. Measurement of Starch and Nitrogen Content

At 7, 14, and 21 days of treatment, rice plants with essentially identical growth conditions were selected, with three replicate measurements conducted for each treatment. The total starch content (TSC) was determined using the Total Starch Assay Kit (Suzhou Comin Biotechnology Co., Ltd., Suzhou, China) following the protocol provided with the kit. To measure the amylose content (AC), 100 mg of rice flour was mixed with 1 mL of 95% ethanol and 9 mL of 1 M NaOH, and then the mixture was boiled for 10 min. After cooling, we diluted the mixture to 100 mL with distilled water. To 5 mL of this solution, we added 1 mL of 1 M aqueous acetic acid solution and 2 mL of iodine solution (0.2 g iodine and 2.0 g potassium iodide in 100 mL aqueous solution). Then, we diluted it to 100 mL with distilled water and measured the absorbance of the solution at 620 nm using a spectrophotometer. The amylopectin content (APC) was calculated by subtracting the amylose content from the total starch content. Nitrogen content was determined using an automatic Kjeltec 8200 instrument (Foss, Hillerød, Denmark) [43].

#### 4.3.4. Yield and Yield Components

Once they reached the mature stage, three pots of uniformly grown rice plants were selected from each treatment. We evaluated the number of panicles per pot, grains per panicle, seed-setting rate, and 1000-grain weight. The actual yield was adjusted to 14% grain moisture content.

#### 4.3.5. Measurement of Taste Value

At the mature stage, the taste value of rice was measured using STA1B-RHS1A equipment (Satake Corp, Hiroshima, Japan). We accurately weighed 30 g of polished rice and placed it into a STA1B stainless steel cup. This was rinsed repeatedly with clean water until thoroughly washed. We added distilled water to the rice–water mixture until it reached 72 g, then covered it with filter paper and soaked it for 30 min. Then, we steamed it for 30 min and kept it warm for 10 min. After the rice was cooked, we gently stirred the rice in the jar, covered it with the filter paper lid, and placed it in a cooling device until it cooled. We took 8 g of rice from the device, placed it in a stainless steel sample ring with a diameter of 30 mm and a height of 9 mm, and used a rice press to prepare the test sample (repeated 3 times). We placed the sample into a measurement chamber and inserted it into the measuring device inside the apparatus. We conducted evaluations on both sides of the rice separately to determine the taste value indicators.

#### 4.3.6. Measurement of Processing Quality and Appearance Quality

We used a Yamamoto Impeller Type Husker (FC-2K, Yamamoto, Suzhou, China) to process the brown rice, which represented the percentage of the whole brown rice weight in the total grain weight. The whole brown rice grains were further processed for about 60 s using a Yamamoto Whitener (VP-32T, Yamamoto, Japan) to obtain head rice grains. The whole-head rice rate refers to the whole-head rice weight percentage in the total grain weight. The chalkiness refers to the percentage of the chalky area in a projected rice grain area, while the chalky grain rate represents the percentage of chalky grains in the total number of head rice grains, which were both determined using a Rice Inspector (ES-1000, Shizuoka, Japan).

### 4.4. Statistical Analysis

Data processing was carried out using Excel 2023, and statistical analysis was performed using SPSS 24.0 software (SPSS Inc., Chicago, IL, USA). A two-way ANOVA was employed with two factors: temperature (normal temperature and high-temperature stress) and different panicle fertilizer application rates (0, 0.34, 0.48, and 0.62 g pot^−1^). All data met the assumptions of normality and homogeneity of variance. Duncan’s one-way ANOVA was used for statistical analysis among the three treatment groups, followed by multiple comparisons (*p* < 0.05). In addition, other data were analyzed using independent *t*-tests (*p* < 0.05). The circular bar plot was generated using the “Circleize” package in R v3.5. Perform correlation analysis using the “linkET” package. Finally, Origin 2022 was used to draw other figures.

## 5. Conclusions

High temperatures during the early grain filling stage reduce the net photosynthetic rate and chlorophyll synthesis in rice leaves, including a decrease in SPAD values, in addition to declines in seed-setting rate and 1000-grain weight, ultimately leading to reduced yield. In addition, high temperatures lead to a decrease in the milled rice rate and head rice rate, while concurrently increasing chalkiness and the chalky grain rate, thereby reducing rice quality. However, with the addition of nitrogen fertilizer at the grain filling stage, normal and high nitrogen application levels mitigated the negative impact of high temperatures on rice yield. Under both normal- and high-nitrogen application conditions, compared with AK, J 606 exhibited less pronounced declines in Pn and SPAD under high-temperature conditions, maintaining a certain level of photosynthetic capacity. Moreover, the activities of NR, GS, and GOGAT in nitrogen metabolism exhibited smaller declines, demonstrating a relatively strong nitrogen assimilation capacity and maintaining certain nitrogen levels. The changes in Rubisco activity in the leaves further demonstrate an improvement in photosynthetic capacity. Among them, the declines in AGPase, GBSS, SBE, and DBE activities in J 606 grains were relatively small. The increase in carbon metabolism is beneficial for improving the sensory quality of rice, thereby maintaining the stability of its palatability. The decrease in TSC, AC, and APC in the grains is relatively small, which aids in preserving sensory quality. In summary, the differences in carbon and nitrogen metabolism affect starch synthesis in grains, ultimately influencing rice quality. Under varying temperature and nitrogen fertilizer conditions, different rice varieties exhibit similar trends in yield changes. In conclusion, cultivating high-quality rice varieties with stable taste properties combined with nitrogen fertilizer application can effectively mitigate high-temperature stress. The authors of future studies should elucidate the physiological and molecular mechanisms underlying the stable taste characteristics of these superior rice varieties under nitrogen fertilization conditions.

## Figures and Tables

**Table 1:** Effects of temperature and nitrogen fertilizer on yield and yield components of AK and J 606.

Variety	Treatment	Effective Panicle Number (/Pot)	Spikelets per Panicle	Seed Setting Rate (%)	1000-Grain Weight (g)	Yield (g/Pot)
AK	HTZ	17.00 ± 0.82 Aa	108.40 ± 4.79 Ac	97.20 ± 0.66 Aa	21.50 ± 0.12 Aa	62.16 ± 3.33 Ad
	NTZ	16.30 ± 0.47 Aa	99.20 ± 7.79 Ac	92.70 ± 2.58 Aa	24.60 ± 0.40 Ba	66.03 ± 2.30 Ac
	HTL	17.70 ± 1.25 Aa	121.40 ± 6.23 Ab	93.40 ± 3.61 Aa	20.00 ± 0.83 Aa	77.87 ± 4.24 Ac
	NTL	17.30 ± 1.25 Aa	116.70 ± 7.76 Ab	94.00 ± 1.75 Aa	23.90 ± 0.14 Bab	81.57 ± 3.61 Ab
	HTN	19.00 ± 0.82 Aa	129.10 ± 3.98 Aa	92.40 ± 4.11 Aa	20.00 ± 1.11 Aab	87.15 ± 4.59 Ab
	NTN	16.70 ± 0.94 Aa	138.10 ± 5.96 Aa	91.60 ± 0.68 Aa	22.70 ± 1.52 Aab	91.79 ± 3.82 Aa
	HTH	20.30 ± 0.94 Aa	144.30 ± 5.99 Aa	92.20 ± 2.27 Aa	18.30 ± 1.15 Ab	96.05 ± 4.84 Aa
	NTH	18.70 ± 1.25 Aa	144.90 ± 6.06 Aa	90.30 ± 3.19 Aa	21.30 ± 0.62 Bb	100.28 ± 4.55 Aa
J 606	HTZ	16.30 ± 1.25 Aa	116.10 ± 9.06 Ac	95.40 ± 1.53 Aa	23.80 ± 0.97 Aa	65.87 ± 3.86 Ac
	NTZ	16.70 ± 0.47 Aa	106.10 ± 5.52 Ab	95.40 ± 0.62 Aa	23.10 ± 0.45 Aa	69.23 ± 4.45 Ac
	HTL	17.00 ± 1.63 Aa	119.80 ± 6.37 Ab	95.10 ± 1.34 Aa	23.60 ± 1.18 Aa	86.15 ± 5.17 Ab
	NTL	17.30 ± 1.70 Aa	127.90 ± 2.58 Ab	95.50 ± 0.24 Aa	22.30 ± 0.51 Aa	89.54 ± 5.26 Ab
	HTN	18.70 ± 1.70 Aa	128.30 ± 5.95 Aa	93.10 ± 1.68 Aa	22.10 ± 0.87 Aa	93.85 ± 5.17 Aa
	NTN	16.70 ± 1.25 Aa	148.60 ± 8.98 Aab	93.50 ± 0.57 Aa	21.90 ± 0.99 Aa	97.72 ± 5.06 Aa
	HTH	19.00 ± 0.82 Aa	137.70 ± 7.83 Aa	93.00 ± 0.38 Aa	21.60 ± 1.08 Aa	101.35 ± 4.92 Aa
	NTH	19.00 ± 2.16 Aa	148.60 ± 4.40 Aa	89.90 ± 5.42 Aa	21.60 ± 0.50 Aa	105.12 ± 5.34 Aa

Note: Values are presented as means ± SD, n = 3. The same uppercase and lowercase letters indicate non-significance, while different uppercase and lowercase letters indicate differences at the level of *p* < 0.05.

**Table 2:** Effects of temperature and nitrogen fertilizer on appearance quality and processing quality of AK and J 606.

Variety	Treatment	Milled Rice Rate (%)	Brown Rice Rate (%)	Head Milled Rice Rate (%)	Chalkiness Degree (%)	Chalky Grain Percentage (%)
AK	HTZ	78.30 ± 0.55 Ac	66.50 ± 0.49 Ac	54.60 ± 0.63 Ad	9.60 ± 0.23 Aa	21.70 ± 0.61 Aa
	NTZ	79.20 ± 0.56 Ad	67.00 ± 0.63 Ad	55.70 ± 0.43 Ad	8.40 ± 0.47 Ba	20.90 ± 0.57 Aa
	HTL	79.20 ± 0.69 Ac	67.20 ± 0.44 Ac	56.30 ± 0.39 Bc	7.20 ± 0.64 Ab	16.30 ± 0.25 Ac
	NTL	80.70 ± 0.60 Ac	68.00 ± 0.43 Ac	58.10 ± 0.55 Ac	6.40 ± 0.30 Ac	16.00 ± 0.35 Ac
	HTN	81.10 ± 0.36 Bb	69.30 ± 0.40 Bb	58.30 ± 0.25 Bb	7.70 ± 0.55 Ab	17.40 ± 0.27 Ab
	NTN	82.40 ± 0.51 Ab	70.50 ± 0.25 Ab	60.60 ± 0.68 Ab	7.00 ± 0.36 Abc	16.90 ± 0.54 Abc
	HTH	83.70 ± 0.64 Ba	71.20 ± 0.33 Aa	61.00 ± 0.36 Ba	7.80 ± 0.40 Ab	18.20 ± 0.48 Ab
	NTH	85.50 ± 0.57 Aa	72.20 ± 0.39 Aa	63.30 ± 0.63 Aa	7.40 ± 0.36 Ab	17.60 ± 0.33 Ab
J 606	HTZ	80.00 ± 0.36 Bd	67.70 ± 0.25 Ad	55.30 ± 0.25 Bd	10.40 ± 0.36 Aa	19.60 ± 0.40 Aa
	NTZ	81.60 ± 0.48 Ad	68.00 ± 0.06 Ad	56.70 ± 0.38 Ad	9.40 ± 0.44 Aa	18.60 ± 0.46 Aa
	HTL	81.50 ± 0.55 Bc	68.50 ± 0.39 Bc	57.30 ± 0.39 Bc	8.80 ± 0.28 Ab	17.10 ± 0.41 Ab
	NTL	83.90 ± 0.38 Ac	69.70 ± 0.38 Ac	59.60 ± 0.24 Ac	8.10 ± 0.30 Ab	16.60 ± 0.54 Ab
	HTN	84.10 ± 0.47 Bb	71.70 ± 0.06 Bb	60.40 ± 0.27 Bb	7.00 ± 0.38 Ac	14.40 ± 0.42 Ab
	NTN	86.30 ± 0.46 Ab	72.80 ± 0.06 Ab	63.30 ± 0.12 Ab	6.70 ± 0.23 Ac	14.10 ± 0.36 Ac
	HTH	86.80 ± 0.54 Ba	73.20 ± 0.06 Aa	63.00 ± 0.44 Ba	7.40 ± 0.49 Ac	15.70 ± 0.54 Ac
	NTH	89.00 ± 0.53 Aa	74.40 ± 0.31 Aa	65.60 ± 0.35 Aa	7.20 ± 0.49 Ac	15.50 ± 0.52 Ac

Note: Values are presented as means ± SD, n = 3. The same uppercase and lowercase letters indicate non-significance, while different uppercase and lowercase letters indicate differences at the level of *p* < 0.05.

**Table 3:** Definitions of different treatments.

Temperature Treatment	N Application Rate for Panicle Fertilizer	Abbreviation
Normal temperature (day/night: 28 °C/18 °C)	Zero N (0 g pot^−1^)	NTZ
	Low N (0.34 g pot^−1^)	NTL
	Normal N (0.48 g pot^−1^)	NTN
	High N (0.62 g pot^−1^)	NTH
High temperature (day/night: 34 °C/24 °C)	Zero N (0 g pot^−1^)	HTZ
	Low N (0.34 g pot^−1^)	HTL
	Normal N (0.48 g pot^−1^)	HTN
	High N (0.62 g pot^−1^)	HTH

## Data Availability

The original contributions presented in the study are included in the article; further inquiries can be directed to the corresponding author.

## References

[1] Guo X., Huang B., Zhang H., Cai C., Li G., Li H., Zhang Y., Struik P.C., Liu Z., Dong M. (2022). T-FACE studies reveal that increased temperature exerts an effect opposite to that of elevated CO_2_ on nutrient concentration and bioavailability in rice and wheat grains. Food Energy Secur..

[2] IPCC (2022). Climate Change 2022: Impacts, Adaptation, and Vulnerability. Contribution of Working Group II to the Sixth Assessment Report of the Intergovernmental Panel on Climate Change.

[3] Stocker T., Qin D., Plattner G., Tignor M., Allen S., Boschung J., Nauels A., Xia Y., Bex V., Midgley P. (2014). Climate Change 2013: The Physical Science Basis.

[4] Wang W., Cai C., He J., Gu J., Zhu G., Zhang W., Zhu J., Liu G. (2020). Yield, dry matter distribution and photosynthetic characteristics of rice under elevated CO_2_ and increased temperature conditions. Field Crops Res..

[5] Shi W., Yin X., Struik P.C., Solis C., Xie F., Schmidt R.C., Huang M., Zou Y., Ye C., Jagadish S.K.J. (2017). High day- and night-time temperatures affect grain growth dynamics in contrasting rice genotypes. J. Exp. Bot..

[6] Gaupp F., Hall J., Hochrainer-Stigler S., Dadson S. (2020). Changing risks of simultaneous global breadbasket failure. Nat. Clim. Change.

[7] Hu Q., Wang W., Lu Q., Huang J., Peng S., Cui K. (2021). Abnormal anther development leads to lower spikelet fertility in rice (*Oryza sativa* L.) under high temperature during the panicle initiation stage. BMC Plant Biol..

[8] Schaarschmidt S., Lawas L.M.F., Kopka J., Jagadish S.K., Zuther E. (2021). Physiological and molecular attributes contribute to high night temperature tolerance in cereals. Plant Cell Environ..

[9] Gourdji S.M., Sibley A.M., Lobell D.B. (2013). Global crop exposure to critical high temperatures in the reproductive period: Historical trends and future projections. Environ. Res. Lett..

[10] Lyman N.B., Jagadish K.S., Nalley L.L., Dixon B.L., Siebenmorgen T. (2013). Neglecting rice milling yield and quality underestimates economic losses from high-temperature stress. PLoS ONE.

[11] Ceccarelli S., Grando S., Maatougui M., Michael M., Slash M., Haghparast R., Rahmanian M., Taheri A., Al-Yassin A., Benbelkacem A. (2010). Plant breeding and climate changes. J. Agric. Sci..

[12] Mittler R., Blumwald E. (2010). Genetic engineering for modern agriculture: Challenges and perspectives. Annu. Rev. Plant Biol..

[13] Chen Y., Chen H., Xiang J., Zhang Y., Wang Z., Zhu D., Wang J., Zhang Y., Wang Y. (2021). Rice spikelet formation inhibition caused by decreased sugar utilization under high temperature is associated with brassinolide decomposition. Environ. Exp. Bot..

[14] Wu C., Song Y., Qi B., Fahad S. (2023). Effects of asymmetric heat on grain quality during the panicle initiation stage in contrasting rice genotypes. J. Plant Growth Regul..

[15] Xu Y.F., Chu C.C., Yao S.G. (2021). The impact of high-temperature stress on rice: Challenges and solutions. Crop J..

[16] Hu Y.J., Xue J.T., Li L., Cong S.M., Yu E.W., Xu K., Zhang H.C. (2021). Influence of dynamic high temperature during grain filling on starch fine structure and functional properties of semi-waxy japonica rice. J. Cereal Sci..

[17] Wang Y., Zhang Y., Shi Q., Chen H., Xiang J., Hu G., Chen Y., Wang X., Wang J., Yi Z. (2020). Decrement of Sugar Consumption in Rice Young Panicle Under High Temperature Aggravates Spikelet Number Reduction. Rice Sci..

[18] Zhen F., Zhou J., Mahmood A., Wang W., Chang X., Liu B., Liu L., Cao W., Zhu Y., Tang L. (2020). Quantifying the effects of short-term heat stress at booting stage on nonstructural carbohydrates remobilization in rice. Crop J..

[19] Tu D., Jiang Y., Salah A., Cai M., Peng W., Zhang L., Li C., Cao C. (2022). Response of Source-Sink Characteristics and Rice Quality to High Natural Field Temperature During Reproductive Stage in Irrigated Rice System. Front. Plant Sci..

[20] Yu X., Wang L., Zhang J., Wang Z., Wang K., Duan Y., Xiao Z., Wang P. (2023). Understanding effects of glutelin on physicochemical and structural properties of extruded starch and the underlying mechanism. Carbohydr. Polym..

[21] Shimoyanagi R., Abo M., Shiotsu F. (2021). Higher temperatures during grain filling affect grain chalkiness and rice nutrient contents. Agronomy.

[22] Arshad M.S., Farooq M., Asch F., Krishna J.S., Prasad P.V., Siddique K.H. (2017). Thermal stress impacts reproductive development and grain yield in rice. Plant Physiol. Biochem..

[23] Chen J., Tang L., Shi P., Yang B., Sun T., Cao W., Zhu Y. (2017). Effects of short-term high temperature on grain quality and starch granules of rice (*Oryza sativa* L.) at post-anthesis stage. Protoplasma.

[24] Yao D., Wu J., Luo Q., Li J., Zhuang W., Xiao G., Deng Q., Lei D., Bai B. (2020). Influence of high natural field temperature during grain filling stage on the morphological structure and physicochemical properties of rice (*Oryza sativa* L.) starch. Food Chem..

[25] Xiong D., Yu T., Ling X., Fahad S., Peng S., Li Y., Huang J. (2015). Sufficient leaf transpiration and nonstructural carbohydrates are beneficial for high-temperature tolerance in three rice (*Oryza sativa*) cultivars and two nitrogen treatments. Funct. Plant Biol.

[26] Wang H.X., Xiong R.Y., Zhou Y.Z., Tan X.M., Pan X.H., Zeng Y.J., Huang S., Shang Q.Y., Xie X.B., Zhang J. (2022). Grain yield improvement in high- quality rice varieties released in southern China from 2007 to 2017. Front. Sustain. Food Syst..

[27] Liu K., Deng J., Lu J., Wang X., Lu B., Tian X., Zhang Y. (2019). High nitrogen levels alleviate yield loss of super hybrid rice caused by high temperatures during the flowering stage. Front. Plant Sci..

[28] Yang J., Chen X., Zhu C., Peng X., He X., Fu J., Ouyang L., Bian J., Hu L., Sun X. (2015). RNA-seq reveals differentially expressed genes of rice (*Oryza sativa*) spikelet in response to temperature interacting with nitrogen at meiosis stage. BMC Genom..

[29] Tang S., Zhang H., Liu W., Dou Z., Zhou Q., Chen W., Wang S., Ding Y. (2019). Nitrogen fertilizer at heading stage effectively compensates for the deterioration of rice quality by affecting the starch-related properties under elevated temperatures. Food Chem..

[30] Kim H.Y., Lim S.S., Kwak J.H., Lee D.S., Lee S.M., Ro H.M., Choi W.J. (2011). Dry matter and nitrogen accumulation and partitioning in rice (*Oryza sativa* L.) exposed to experimental warming with elevated CO_2_. Plant Soil.

[31] Tanaka M., Keira M., Yoon D.K., Mae T., Ishida H., Makino A., Ishiyama K. (2022). Photosynthetic enhancement, lifespan extension, and leaf area enlargement in flag leaves increased the yield of transgenic rice plants overproducing Rubisco under sufficient N fertilization. Rice.

[32] Ling Q.H., Su Z.F., Chang H.C., Cai J.Z., Ho J.S. (1983). The leaf-age model of development process in different varieties of rice. Sci. Agric. Sin..

[33] Nakano H., Makino A., Mae T. (1997). The effect of elevated partial pressures of CO_2_ on the relationship between photosynthetic capacity and N content in rice leaves. Plant Physiol..

[34] Marchiori P.E., Machado E.C., Ribeiro R.V. (2014). Photosynthetic limitations imposed by self-shading in field-grown sugarcane varieties. Field Crops Res..

[35] Li J., Feng Y., Wang X., Xu G., Luo Z., Peng J., Luo Q., Lu W., Han Z. (2022). High nitrogen input increases the total spikelets but decreases the high-density grain content in hybrid indica rice. Field Crops Res..

[36] Wei H., Zhu Y., Qiu S., Han C., Hu L., Xu D., Zhou N., Xing Z., Hu Y., Cui P. (2018). Combined effect of shading time and nitrogen level on grain filling and grain quality in japonica super rice. J. Integr. Agric..

[37] Ambavaram M.M., Basu S., Krishnan A., Ramegowda V., Batlang U., Rahman L., Baisakh N., Pereira A. (2014). Coordinated regulation of photosynthesis in rice increases yield and tolerance to environmental stress. Nat. Commun..

[38] Deng F., Wang L., Pu S.L., Mei X.F., Li S.X., Li Q.P., Ren W.J. (2018). Shading stress increases chalkiness by postponing caryopsis development and disturbing starch characteristics of rice grains. Agric. For. Meteorol..

[39] Li Q., Deng F., Zeng Y., Li B., He C., Zhu Y., Zhou X., Zhang Z., Wang L., Tao Y. (2022). Low light stress increases chalkiness by disturbing starch synthesis and grain filling of rice. Int. J. Mol. Sci..

[40] Stitt M., Zeeman S.C. (2012). Starch turnover: Pathways, regulation and role in growth. Curr. Opin. Plant Biol..

[41] Stitt M., Huber S., Kerr P. (1987). Control of photosynthetic sucrose formation. Photosynthesis.

[42] Li Y., Liu K., Chen F., Cheng Y. (2018). Comparative proteomics analysis reveals the effect of germination and selenium enrichment on the quality of brown rice during storage. Food Chem..

[43] Ma Z., Cao J., Chen X., Yu J., Liu G., Xu F., Hu Q., Li G., Zhu Y., Zhang H. (2025). Differences in carbon and nitrogen metabolism of soft japonica rice in southern China during grain filling stage under different light and nitrogen fertilizer conditions and their relationship with rice eating quality. Front. Plant Sci..

[44] Chen X.Y., Zhu Y., Ma Z.T., Zhang M.Y., Wei H.Y., Zhang H.C., Liu G.D., Hu Q., Li G.Y., Xu F.F. (2023). Effects of light intensity and nitrogen fertilizer interaction on carbon and nitrogen metabolism at grain-filling stage and its relationship with yield and quality of southern soft japonica rice. Acta Agron. Sin..

[45] Forde B.G., Clarkson D.T. (1999). Nitrate and ammonium nutrition of plants: Physiological and molecular perspectives. Advances in Botanical Research.

[46] Krouk G., Crawford N.M., Coruzzi G.M., Tsay Y.F. (2010). Nitrate signaling: Adaptation to fluctuating environments. Curr. Opin. Plant Biol..

[47] Foyer C.H., Valadier M.H., Migge A., Becker T.W. (1998). Drought-induced effects on nitrate reductase activity and mRNA and on the coordination of nitrogen and carbon metabolism in maize leaves. Plant Physiol..

[48] Plaut Z. (1974). Nitrate reductase activity of wheat seedlings during exposure to and recovery from water stress and salinity. Physiol. Plant.

[49] Lawlor D.W. (2002). Limitation to photosynthesis in water-stressed leaves: Stomata vs. metabolism and the role of ATP. Ann. Bot..

[50] Masclaux-Daubresse C., Reisdorf-Cren M., Pageau K., Lelandais M., Grandjean O., Kronenberger J., Valadier M.H., Feraud M., Jouglet T., Suzuki A. (2006). Glutamine synthetase-glutamate synthase pathway and glutamate dehydrogenase play distinct roles in the sink-source nitrogen cycle in tobacco. Plant Physiol..

[51] Sánchez-Rodríguez E., del Mar Rubio-Wilhelmi M., Ríos J.J., Blasco B., Rosales M.Á., Melgarejo R., Romero L., Ruiz J.M. (2011). Ammonia production and assimilation: Its importance as a tolerance mechanism during moderate water deficit in tomato plants. J. Plant Physiol..

[52] Konishi N., Ishiyama K., Matsuoka K., Maru I., Hayakawa T., Yamaya T., Kojima S. (2014). NADH-dependent glutamate synthase plays a crucial role in assimilating ammonium in the Arabidopsis root. Physiol. Plant.

[53] Liang C., Chen L., Yan W., Jia L., Xu G., Tian L. (2011). High temperature at grain-filling stage affects nitrogen metabolism enzyme activities in grains and grain nutritional quality in rice. Rice Sci..

[54] Zhou L., Liang S., Ponce K., Marundon S., Ye G., Zhao X. (2015). Factors affecting head rice yield and chalkiness in indica rice. Field Crops Res..

[55] Zhang J., Zhang Y., Song N., Chen Q., Sun H., Peng T., Huang S., Zhao Q. (2021). Response of grain-filling rate and grain quality of mid-season indica rice to nitrogen application. J. Integr. Agric..

[56] Chen T., Wilson L.T., Liang Q., Xia G., Chen W., Chi D. (2017). Influences of irrigation, nitrogen and zeolite management on the physicochemical properties of rice. Arch. Agron. Soil Sci..

[57] Zhang H., Ke X., Hui G. (2017). Effects of nitrogen level on yield and quality of japonica soft super rice. J. Integr. Agric..

[58] Dhillon A.K., Sharma N., Dosanjh N.K., Goyal M., Mahajan G. (2018). Variation in the nutritional quality of rice straw and grain in response to different nitrogen levels. J. Plant Nutr..

[59] Wang C., Jing W., Fa X., Gu J., Wang W., Zhu K., Zhang W., Gu J., Liu L., Wang Z. (2025). Effect of nitrogen levels on grain yield and quality in improving mid-season japonica rice varieties. J. Sci. Food Agric..

[60] Guo C., Zhang L., Jiang P., Yang Z., Chen Z., Xu F., Guo X., Sun Y., Ma J. (2024). Grain chalkiness is decreased by balancing the synthesis of protein and starch in hybrid indica rice grains under nitrogen fertilization. Foods.

[61] Dou Z., Zhou Y., Zhang Y., Guo W., Xu Q., Gao H. (2024). Influence of nitrogen applications during grain-filling stage on rice (*Oryza sativa* L.) yield and grain quality under high temperature. Agronomy.

[62] Ye C., Ma H., Huang X., Xu C., Chen S., Chu G., Zhang X., Wang D. (2022). Effects of increasing panicle-stage N on yield and N use efficiency of indica rice and its relationship with soil fertility. Crop J..

